# Surface Electromyography-Controlled Automobile Steering Assistance

**DOI:** 10.3390/s20030809

**Published:** 2020-02-02

**Authors:** Edric John Cruz Nacpil, Kimihiko Nakano

**Affiliations:** Institute of Industrial Science, The University of Tokyo, 4-6-1 Komaba, Meguro-ku, Tokyo 153-8505, Japan; knakano@iis.u-tokyo.ac.jp

**Keywords:** automated driving, human-machine interface (HMI), surface electromyography (sEMG), advanced driver assistance system (ADAS)

## Abstract

Disabilities of the upper limb, such as hemiplegia or upper limb amputation, can limit automobile drivers to steering with one healthy arm. For the benefit of these drivers, recent studies have developed prototype interfaces that realized surface electromyography (sEMG)-controlled steering assistance with path-following accuracy that has been validated with driving simulations. In contrast, the current study expands the application of sEMG-controlled steering assistance by validating the Myo armband, a mass-produced sEMG-based interface, with respect to the path-following accuracy of a commercially available automobile. It was hypothesized that one-handed remote steering with the Myo armband would be comparable or superior to the conventional operation of the automobile steering wheel. Although results of low-speed field testing indicate that the Myo armband had lower path-following accuracy than the steering wheel during a 90° turn and wide U-turn at twice the minimum turning radius, the Myo armband had superior path-following accuracy for a narrow U-turn at the minimum turning radius and a 45° turn. Given its overall comparability to the steering wheel, the Myo armband could be feasibly applied in future automobile studies.

## 1. Introduction

Some drivers can experience stroke-induced hemiplegia that paralyzes one side of the body, whereas other drivers have upper limb amputation. Drivers with either of these health conditions have relied on devices such as steering knobs, to enable steering with one healthy arm or a prosthetic limb [1,2]. The advent of automated vehicles eventually led to the consideration of relegating steering to the vehicle for the benefit of persons with disabilities, although vulnerability of vehicle computers to hackers, along with the high cost and susceptibility to severe weather conditions of advanced automation sensors, such as lidar, can compromise the safety of drivers, passengers, and other people in the vehicle environment [3,4,5]. An additional problem with vehicle automation is the possibility of accidents resulting from inappropriate driver input when transitioning from automated driving to manual mode during unexpected scenarios, such as road construction or suddenly approaching road obstacles [6,7,8]. Furthermore, many current automobiles rely primarily on conventional steering wheel interfaces. 

Aside from steering knobs that can be readily mounted on to the rims of steering wheels, other devices such as joysticks, motion sensors, and strain gauges have been developed [3,9,10]. However, a problem that such sensors have in common is the requirement of force input that may not be readily available from drivers with amputated upper limbs. Although some drivers could use healthy upper limbs or adapt steering knobs to interface with prosthetic upper limbs, other potential steering interfaces such as brain–computer interface (BCI) sensors or eye-gaze tracking sensors require no force input from the driver, and thus require less physical effort to operate [1]. However, one major challenge for eye-gaze tracking is the “Midas touch problem” by which camera sensors are unable to distinguish between eye movement for control commands and eye movement for visual information gathering [11]. It has also been observed that BCIs utilizing electroencephalography (ECG) sensors can be difficult, if not impossible, to operate by about 20% of users with “BCI illiteracy” [12,13]. In contrast, no such usability problem has been widely observed for interfaces utilizing surface electromyography (sEMG) sensors to measure electrical muscle activity. Furthermore, sEMG interfaces can rely on muscle activation thresholds or muscle pattern recognition algorithms to distinguish between muscle activities for command inputs and other muscle activities [14,15,16].

Although sEMG sensors have commonly been used to control devices such as prosthetic limbs and to evaluate driver-related information such as steering comfort, sEMG has been considered in recent years by a handful of studies, as an alternative steering input signal to enable remote steering wheel control [17,18,19,20,21,22,23,24]. In two previous driving simulator experiments, investigators of the current study developed prototype interfaces that relied on self-adhesive wet sEMG electrodes to measure muscle activity [25,26]. The path-following accuracy of the interfaces was measured by having test drivers complete driving simulations of low-speed turning maneuvers. Results of the driving simulations indicated that the sEMG-based interfaces were comparable overall to steering wheel-type interfaces with respect to 45° turns, 90° turns, and wide U-turns at twice the minimum turning radius of the simulated automobile. In the case of a narrow U-turn at the minimum turning radius, the sEMG-based interfaces were significantly superior to the steering wheel-type interfaces. Another advantage of one of the previous interfaces was the ability of sEMG electrodes to be easily reconfigured for amputees. This interface relied on isometric contractions of the biceps brachii that have been used as control input for some powered prosthetic upper limbs [27]. The Myo armband proposed by the current study could also be modified to receive sEMG input from an amputated upper limb to control a prosthesis [28,29]. Therefore, unlike joysticks, motion sensors, or other devices that rely on the movement of existing members of the upper limb, the Myo armband for the steering assistance system in the current study has the potential to measure muscle activity, even if some or all of the upper limb is unavailable.

Since the ability of a vehicle to adhere to a desired path is necessary to avoid collision accidents with surrounding objects, the path-following accuracy of the Myo armband was measured, as with the previous driving simulator experiments conducted by the investigators. However, one major difference from these experiments is the use of an actual automobile, in order to avoid driving simulation drawbacks such as simulator motion sickness and reduced perceptual fidelity to actual driving [30]. Another major difference from the previous experiments is the reliance of the mass-produced Myo armband on dry electrodes that are mounted on the forearm with an elastic band, thereby eliminating the need for electrode adhesive or the conductive gel of wet electrodes. Hence, the current study significantly contributes to the previous driving simulator experiments by confirming the path-following accuracy of a sEMG-controlled steering assistance with an actual automobile and a mass-produced sEMG-based interface. 

Based on the previous driving simulator studies conducted by the investigators, it was hypothesized that the Myo armband would have comparable or superior path-following accuracy in comparison to the steering wheel of the actual automobile. Given that the Myo armband was comparable overall to the steering wheel, the feasibility of the Myo armband as a steering assistance interface was confirmed. The rest of this paper details the steps leading to this conclusion as follows: Section 2 concerns the materials and methods for the experiment with the actual vehicle, whereas the results are presented in Section 3. The results are discussed in Section 4, and finally the conclusions of the study are provided in Section 5.

## 2. Materials and Methods

Experimental equipment and methodology for the current study are described as follows: Section 2.1 concerns the sEMG-based steering assistance interface and Section 2.1.1 describes the setup of global positioning system (GPS) equipment to measure the motion of the test vehicle. Section 2.2 concludes the chapter by explaining the procedures for the experimental trials with an actual automobile and subsequent data processing.

### 2.1. Steering Assistance Interface

**Figure 1:**
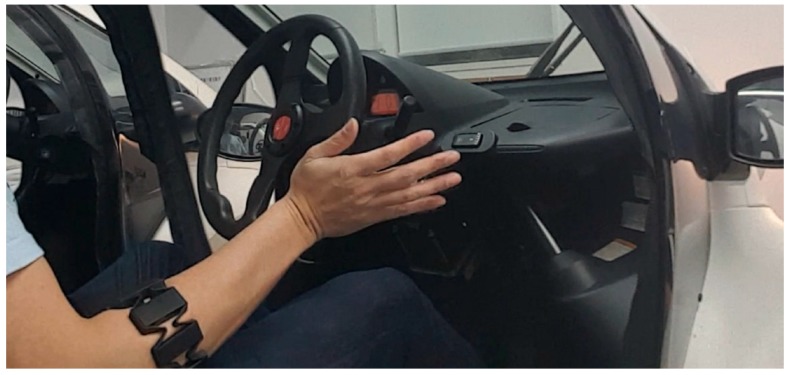
Myo armband used on right forearm to control steering wheel of COMS automobile.

Since the COMS B•COM, a small-scale electric automobile by Toyota Auto Body Co. that is henceforth referred to as the ‘COMS’, has been employed in other studies to develop steering systems, the COMS is a suitable platform for developing an sEMG-controlled interface, as shown in Figure 1 [31,32,33,34]. The measurement of sEMG was realized with the Myo armband, a commercially available sEMG interface device that was introduced in 2013 by Thalmic Labs, Inc., Waterloo, ON, Canada [24]. Although the potential of the Myo armband as method for automotive steering had not been previously investigated, researchers recognized the potential of the armband beyond its original purpose as an entertainment device by successfully controlling devices for daily living such as robotic assistants and prosthetic limbs [35,36,37]. Relative to similar mass-produced sEMG equipment, the Myo armband was chosen as an affordable and accurate device to assess the feasibility of mass-produced sEMG equipment for steering assistance [38]. 

**Figure 2:**
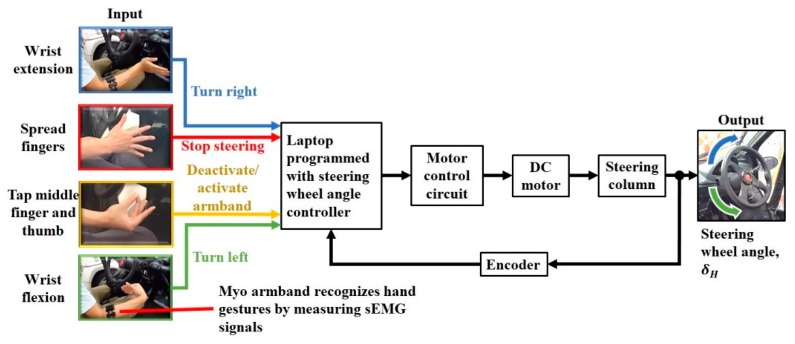
Steering assistance control scheme.

The Myo armband was worn on the right forearm to recognize a set of default hand gestures, based on forearm sEMG signals recorded by eight stainless steel electrodes at 200 Hz (Figure 2) [39]. Calibration for these gestures was performed for each user with the software from the manufacturer. The software also allows the gestures to be mapped to custom keyboard strokes that were converted by the laptop into steering wheel rotation. For example, wrist flexion of the right hand rotated the steering wheel leftward, whereas wrist extension of the same hand resulted in rightward rotation. Spreading the fingers of the right arm stopped steering wheel rotation, and in order to free the right arm for other objectives besides remote steering, rapidly tapping the middle figure and thumb together twice toggled between deactivation and reactivation of the Myo armband. 

The Myo armband wirelessly transmits detection signals for hand gestures to a PC platform laptop (N17C1, Acer, Inc., Taipei, Taiwan) that implements a steering wheel angle controller through a custom C# program. Based on the type of hand gesture, the steering wheel angle controller sends DC motor commands to a motor control circuit (Controller BLV620K200S-3, Oriental Motor Co., Ltd., Tokyo, Japan). A voltage signal is sent from the motor control circuit to a brushless DC motor (Motor BLV620K200S-3, Oriental Motor Co., Ltd., Tokyo, Japan). The DC motor is connected to the steering column of the COMS automobile by a pair of gears with a ratio of 1:1. These gears allow the steering column to be rotated by the DC motor to adjust the steering wheel angle (SWA), *δ_H_*. Based on the measured SWA at 100 Hz from an encoder (SKM36S-HVA0-K02, SICK AG, Waldkirch, Germany) that is driven by the steering column, the steering wheel angle controller determines the difference between measured SWA and the target SWA. The steering wheel angle controller alters the command to the motor control circuit according to the difference, thereby achieving closed-loop SWA control.

**Figure 3:**
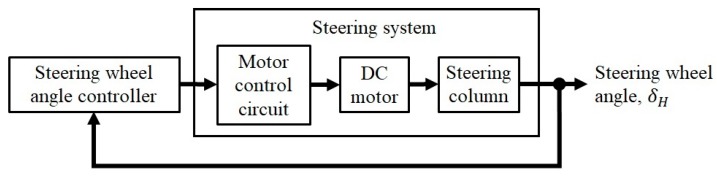
Static steering assistance control system model with steering wheel angle controller and steering system.

The steering system was defined as an assembly consisting of the motor control circuit, DC motor, and steering column (Figure 3). In accordance with a voltage setting command from the steering wheel angle controller, the motor control circuit provided voltage to the DC motor that rotates the steering column. The output of the steering system was the steering wheel angle.

**Figure 4:**
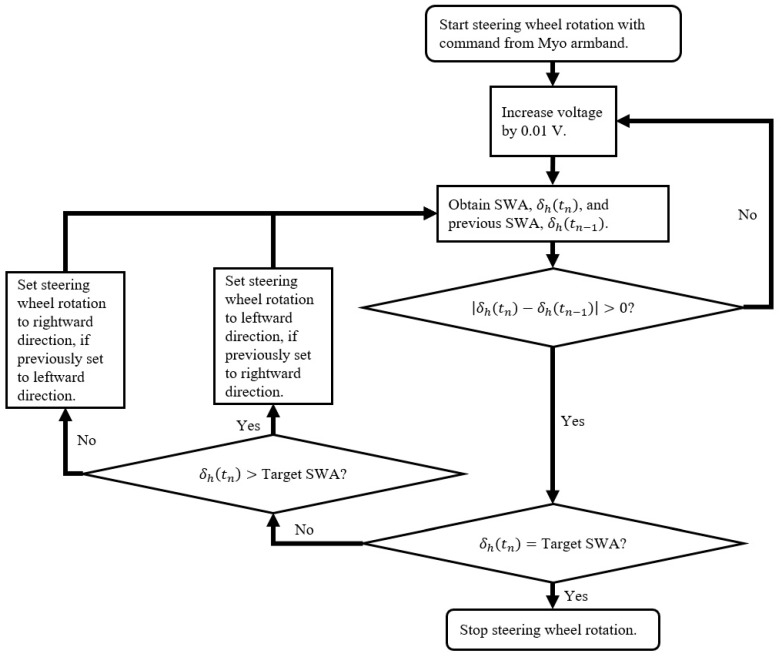
Steering wheel angle control algorithm for rightward steering wheel rotation.

The algorithm was only developed to address steering wheel rotation between 0° and the maximum rightward SWA of 625°, since the COMS only executed right turns in the driving scenarios. When the driver performs wrist extension with the wheel at 0°, the Myo armband recognizes this gesture and sends a command to the steering wheel angle controller to initiate rightward steering wheel rotation. The voltage command sent from the controller to the DC motor control circuit is increased in increments of 0.01 V from an initial value of 0 V (Figure 4). With the exception of the wide U-turn with a radius of curvature equal to twice the minimum turning radius of the COMS, all driving scenarios were designed to be performed with the target SWA being equal to the maximum SWA. In the case of the wide U-turn, trial and error testing determined the target SWA to be equal to 405°. 

As opposed to the commonly applied proportional derivative control, the method shown in Figure 4 gradually increased the rotation speed of the steering wheel to reduce wear on the steering system caused by jerk on the steering column from the DC motor. This jerk was observed by the sudden rotation of steering wheel during preliminary tests. A more important benefit is the reduced risk of injury to the driver from accidentally holding the steering wheel during rotation, since the steering wheel is rotated gradually. Further details on the procedure for evaluating the performance of this algorithm with respect to the steering wheel angle are described in the methodology section of this study. Data from the performance evaluation are contained in the results and discussion section of this study.

#### 2.1.1. Position Tracking Equipment for Test Vehicle

**Figure 5:**
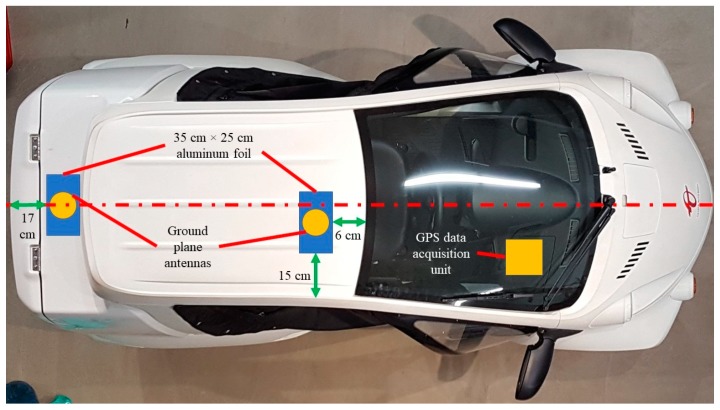
Setup of antennas for GPS data logging system mounted in cabin of COMS vehicle.

In order to measure the position of the COMS during turning maneuvers, a GPS data acquisition unit (Racelogic RLVB2SX) was mounted in the cabin of the COMS. GPS satellite signal transmission with the unit was achieved by mounting and wiring two metal ground plane antennas on top of the vehicle as shown in Figure 5. Since the antennas required a metal mounting surface, and the roof of the COMS was not made of metal, aluminum foil sheets were placed between the top of the vehicle and the antennas. The position, speed, and lateral acceleration of the vehicle were recorded at 20 Hz. Position accuracy was ±20 cm during optimal satellite communication. 

The ground plane antenna closest to the rear of the COMS was the primary antenna through which all vehicle motion parameters were calculated. Since vehicle position, speed, and lateral acceleration were the only parameters examined, it was not necessary to measure pitch and roll by aligning the primary and secondary ground plane antennas along the longitudinal axis of the vehicle, i.e., the dashed red line in Figure 5.

### 2.2. Methodology

In accordance with the Declaration of Helsinki and with permission from the Ethics Committee of the Interfaculty Initiative in Information Studies, Graduate School of Interdisciplinary Information Studies, The University of Tokyo (No. 15 in 2018), five male test subjects with an average age of 36 SD (standard deviation) 13 and an average of 15.4 SD 11.5 years of driving experience were recruited as test drivers. All subjects provided informed consent prior to participating in the experiment.

**Figure 6:**
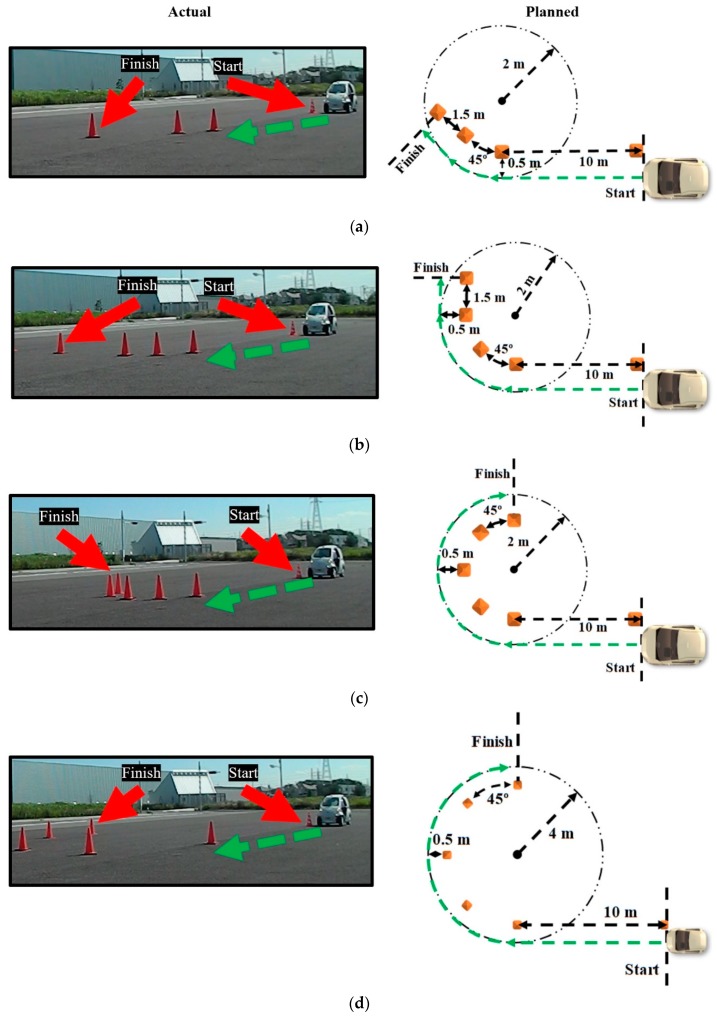
All drivers tested path-following accuracy of steering wheel and Myo armband by performing: (**a**) 45° turn; (**b**) 90° turn; (**c**) narrow U-turn; and (**d**) wide U-turn.

Since the experiment was a means to determine the extent to which the results of previous driving simulator studies conducted by the investigators were applicable to an actual automobile, the driving scenarios were patterned after the driving simulations in [25,26], as illustrated in Figure 6. The test drivers performed several driving scenarios: a 45° turn, 90° turn, narrow U-turn with a radius of curvature equal to the minimum turning radius of 2 m for the COMS, and a wide U-turn with a radius of curvature equal to the twice the minimum turning radius. 

Path following accuracy for the steering wheel and the Myo armband was defined as the average lateral error across all drivers. The average lateral error was measured as the shortest distance between the centerline of the vehicle and the ideal trajectory of each driving scenario. The ideal trajectory is the path followed by the centerline of the vehicle by driving as close as possible to the road cones without touching the edges of the road cone bases that face away from the center of the turning circle. Hence, drivers were instructed to drive as close as possible to the road cones without touching them. Based on previous driving simulator studies in [25,26], it was hypothesized that the path-following accuracy the Myo armband would be comparable or superior to the steering wheel.

In preparation for the experiment, the Myo armband was calibrated for each driver with the proprietary software from the Myo armband manufacturer. The software was preprogrammed by the manufacturer to record and map each of the hand gestures shown in Figure 2. Software settings enabled the gestures to be mapped to individual computer keystrokes on the laptop that executed the SWA control algorithm. Based on these keystrokes, the algorithm enabled remote steering wheel operation.

Previous testing demonstrated that the Myo armband had a maximum mean gesture classification percent error of 11.95 SD 9.00. There was no significant difference between this error and the error of 12.48 SD 8.51 for conventional sEMG equipment with disposable Ag/AgCl bipolar electrodes [39].

Each driver performed each of the four steering maneuvers in Figure 6 twice: once with the steering wheel and a second time with the Myo armband interface. Therefore, there were eight experimental conditions per driver. In order to train for these conditions, the drivers performed one practice run immediately before each of the actual experimental conditions. In order to minimize learning effects from training and the actual experiment, within subject randomization with a balanced Latin square determined the order in which the experimental conditions were performed. 

VBOX File Processor software was used to convert the GPS data, including position, speed, and lateral acceleration, into two-dimensional cartesian coordinates that were read as CSV files into Microsoft Excel. Then the position data was adjusted in Excel to be plotted on 2-D planes that provide a graphical representation of the turning trajectories. Only the automatic curve generation feature in Excel was used to produce the plots. Graphs for speed and lateral acceleration were also generated. All of the graphical data would be used to understand the path-following accuracy associated with each steering interface. 

Since *p* < 0.05 for the Shapiro–Wilk test for normality for each driving scenario, the null hypothesis that the average trajectory for each scenario was normally distributed was rejected. Hence, the nonparametric Wilcoxon sign rank test was used, in order to determine whether or not there was any significant difference in average lateral error between the Myo armband and the COMS steering wheel. For the Wilcoxon signed rank test, *p* < 0.05 to reject the null hypothesis that there is no significant difference between the steering wheel and the Myo armband with respect to average lateral error. In order for the path-following accuracy of the Myo armband to be confirmed, it would have to be at least comparable overall to the steering wheel. 

Based on the average trajectory data, the average SWA throughout each driving scenario was calculated by multiplying the COMS steering ratio of 14.2:1 by

δ_F_ = *l*/R (1)

The average SWA was plotted as function of time to determine the performance of the steering wheel controller for the Myo armband. The average and maximum steering wheel rate (SWR) was calculated from the SWA for further explanation of the experimental results with respect to SWR and SWA. 

## 3. Results

**Figure 7:**
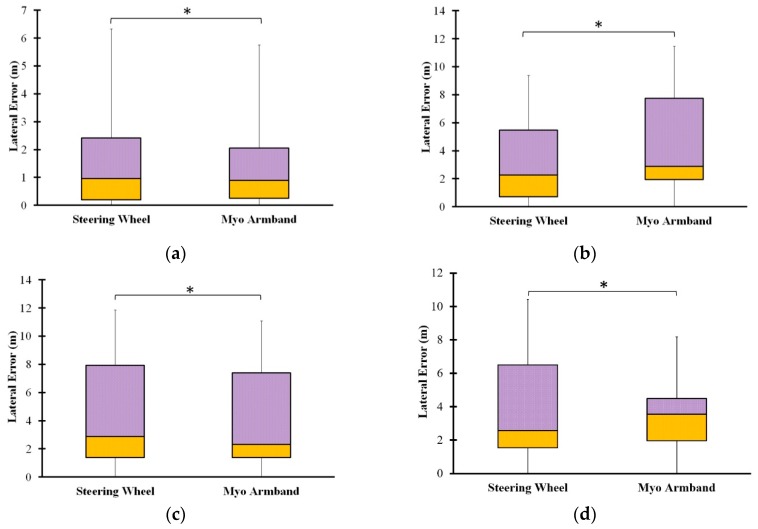
Median lateral errors used to evaluate path-following accuracy of steering wheel and Myo armband in the case of four driving scenarios: (**a**) 45° turn, (**b**) 90° turn, (**c**) narrow U-turn, and (**d**) wide U-turn. * indicates statistically significant difference between averages of lateral error distributions.

Comparison between the steering wheel of the COMS vehicle and the Myo armband was conducted by analyzing measured turning trajectory data from the experimental sessions with the test drivers. With respect to path-following accuracy, Figure 7 indicates that the Myo armband was more accurate than the steering wheel for the 45° turn and the narrow U-turn. However, the steering wheel was more accurate in the case of the 90° turn and the wide U-turn. Given that each interface was superior in two of the four driving scenarios, the path-following accuracy of the Myo armband was confirmed because it was comparable overall to the steering wheel. 

**Figure 8:**
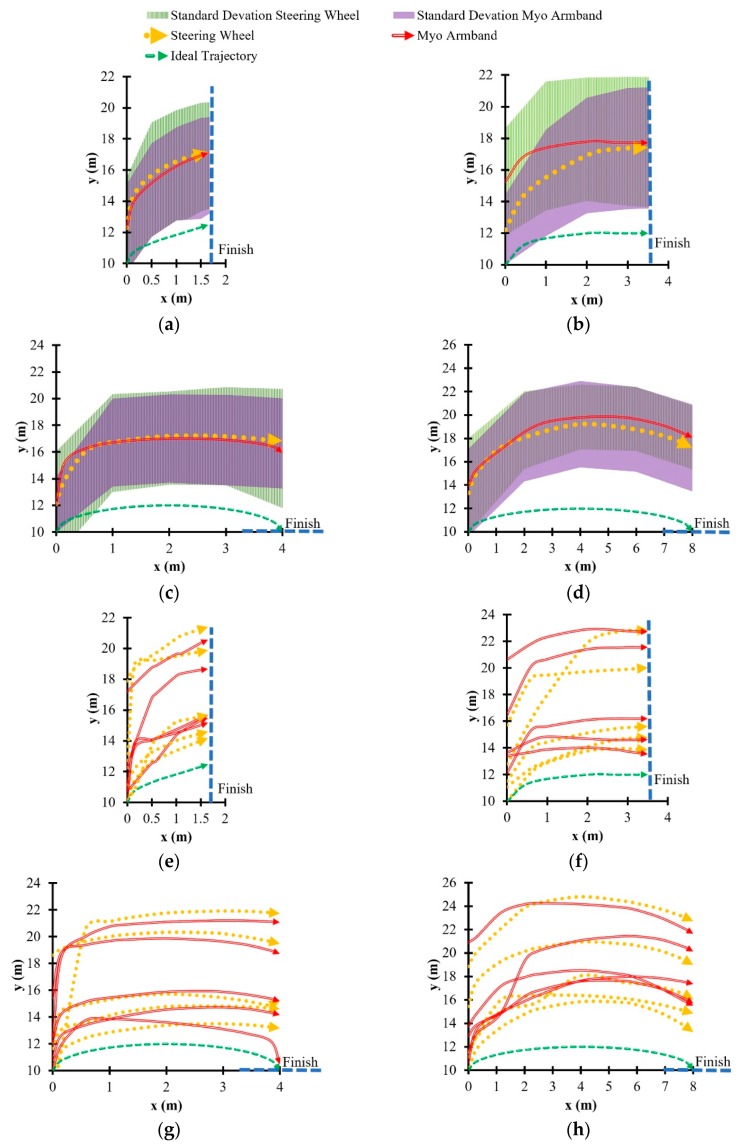
Average turning trajectories of steering wheel and Myo armband resulting from five test drivers performing: (**a**) 45° turn, (**b**) 90° turn, (**c**) narrow U-turn, and (**d**) wide U-turn. Individual turning trajectories for all five drivers shown for (**e**) 45° turn, (**f**) 90° turn, (**g**) narrow U-turn, and (**h**) wide U-turn.

Turning trajectories and their corresponding standard deviations are shown in Figure 8. The standard deviation regions were calculated at five evenly spaced points along each ideal trajectory. Since the current study primarily aimed to assess the feasibility of the Myo armband with respect to average lateral error, rather than the variability of vehicle trajectory, the standard deviation regions are intended to be general approximations. According to the turning trajectories in Figure 8 that were measured after the COMS vehicle passed the second road cone along the target path of each driving scenario, drivers tended to initiate turning later into the 90° turn, when using the Myo armband as opposed to the steering wheel. Hence, the turning trajectory associated with the Myo armband tends to follow the ideal trajectory less accurately than the trajectory associated with the steering wheel. This observation supports the lower path-following accuracy of the Myo armband in the case of the 90° turn, as shown by the box plot in Figure 7b. Contrastingly, in the case of the 45° turn and the narrow U-turn, the trajectories of the Myo armband tend to follow the ideal trajectories more accurately than the trajectories of the steering wheel. Thus, the Myo armband has higher path-following accuracy (Figure 7a,c).

**Figure 9:**
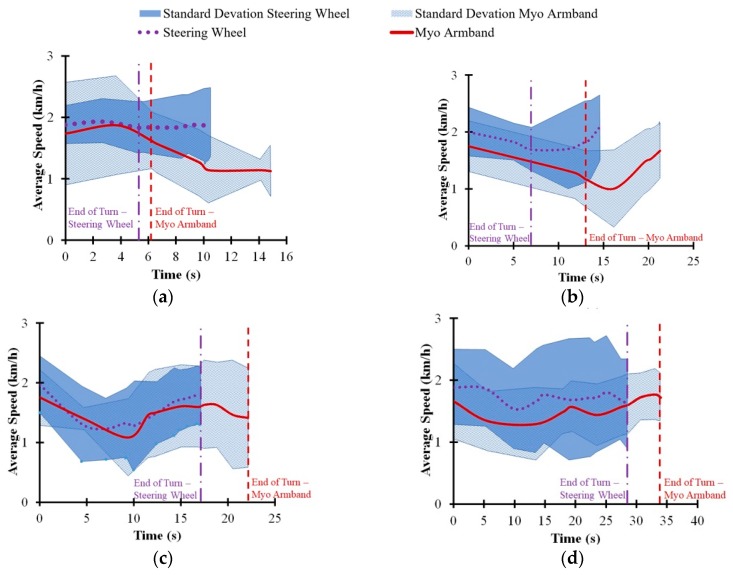
Average vehicle speed associated with steering wheel and Myo armband for (**a**) 45° turn, (**b**) 90° turn, (**c**) narrow U-turn, and (**d**) wide U-turn. Vertical dashed lines indicate average times when drivers exited turns.

**Figure 10:**
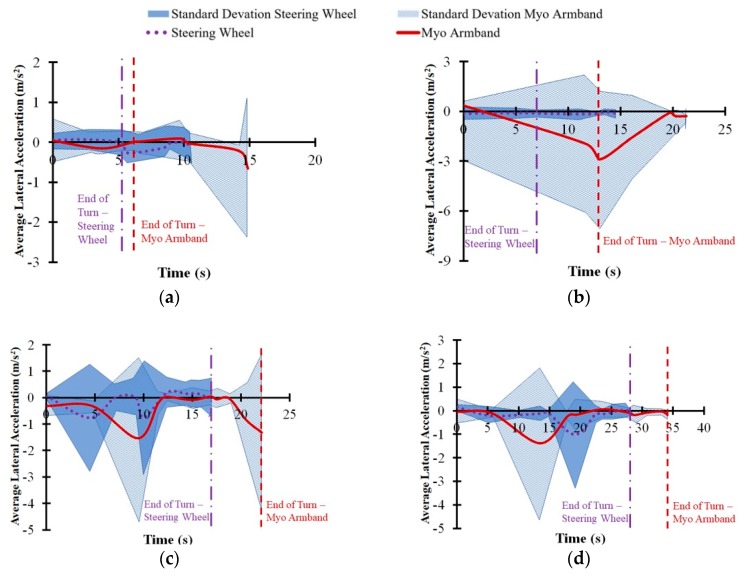
Average lateral acceleration on COMS vehicle for (**a**) 45° turn, (**b**) 90° turn, (**c**) narrow U-turn, and (**d**) wide U-turn. Vertical dashed lines indicate average times when drivers exited turns.

**Figure 11:**
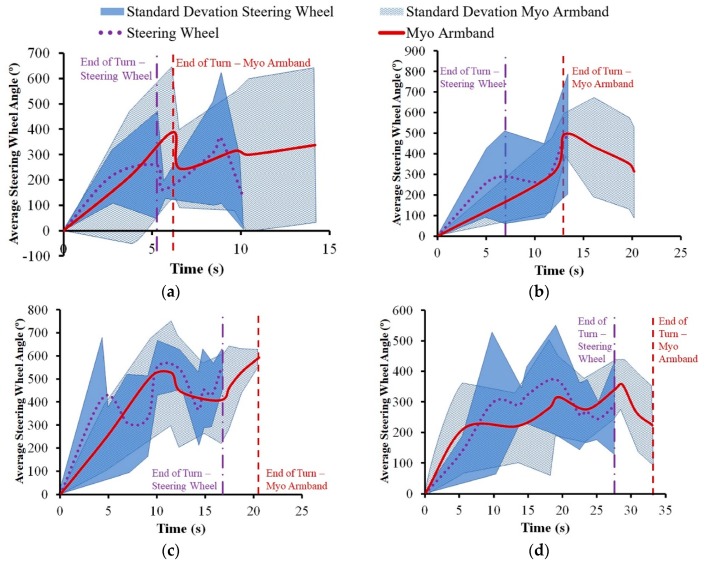
Average steering wheel angle of COMS vehicle for (**a**) 45° turn, (**b**) 90° turn, (**c**) narrow U-turn, and (**d**) wide U-turn. Vertical dashed lines indicate average times when drivers exited turns.

As shown in Figure 9, the difference in vehicle speed between the Myo armband and the steering wheel did not exceed 0.5 km/h throughout each turning maneuver. However, the Myo armband tended to be lower than the steering wheel with respect to the average vehicle speed for the 90° turn and the wide U-turn. There was also another noticeable difference between the interfaces, since the Myo armband tended to have more average lateral acceleration toward the inside of the 90° turn (Figure 10b) and a lower average steering wheel angle for the same turn (Figure 11b). Furthermore, the Myo armband had higher average and maximum SWRs for the 90° turn (Table 1). Given that the Myo armband was less accurate for the 90° turn, significantly lower path-following accuracy was therefore associated with:

- higher maximum and average SWR
- lower average vehicle speed

Explanations for these attributes are provided in the next section.

## 4. Discussion

The Myo armband provided more accurate path following than the steering wheel in the case of the 45° turn, and as predicted by the previous studies involving simulations of the driving scenarios from the current study [25,26], the Myo armband was more accurate in the case of the narrow U-turn. However, with regard to the 90° turn and wide U-turn, the Myo armband was unexpectedly less accurate than the steering wheel. In order to explain these results, Section 4.1 will consider path-following accuracy in relation to vehicle speed, lateral acceleration, SWA, and SWR. Section 4.2 will then discuss the limitations of the conclusions that might be drawn from the results.

### 4.1. Relationship Between Path-Following Accuracy and Vehicle Motion

The tendency of the Myo armband to have lower vehicle speed for the wide U-turn and the 90° turn could indicate a tendency of the drivers to avoid understeer by decelerating the vehicle. Another possibility might be a lack of confidence in the Myo armband. Support for these potential explanations, in the case of the 90° turn, is shown by the trajectories in Figure 8b. Relative to the turning trajectory of the steering wheel, drivers used the Myo armband to initiate the turn at a further distance from the beginning of the 90°turn. According to Figure 8f, two out of the five drivers turned at a noticeably further distance than the other three drivers. Since this distinction applies for both the Myo armband and the steering wheel, it is possible that the drivers, rather than the interfaces caused the delayed turning. Although it is possible that the trajectories of these two drivers could have influenced the average trajectory in Figure 8b, the other individual driver trajectories for the Myo armband also tend to be further from the start line than the corresponding trajectories for the steering wheel. Consequently, the drivers may have decelerated the vehicle to reduce understeer or out of a lack of confidence, as suggested by the decreasing speed indicated by Figure 9b. Previous empirical observations indicate that deceleration along a circular path results in lateral acceleration on the vehicle towards the inside of the turn [40]. Therefore, as expected, Figure 10b suggests that centripetal acceleration resulted from vehicle deceleration. 

Similarly, in the case of the wide U-turn, lower vehicle speed and higher centripetal acceleration can be observed in Figure 9d and Figure 10d, respectively. As in the case of the for 90° turn, the same potential explanations concerning deceleration caused by the drivers may also apply to the U-turn.

Another consequence of decreasing speed is increasing ground reaction force at the front steering tires of the COMS vehicle [41,42]. For conventional steering wheels, more torque input from the driver is required to turn the steering wheel, when ground reaction forces increase. Similarly, the steering wheel angle controller for the armband commands the DC motor to apply more torque to the steering column. As suggested by Figure 9b,d as well as Table 1, the lower vehicle speed associated with using the Myo armband resulted in higher torque input from the DC motor that translated into higher average and maximum SWRs for the Myo armband, as opposed to the steering wheel. Increased SWR for the Myo armband is especially noticeable at the end of the 90° turn (Figure 11b). In contrast, Figure 11b indicates that the SWR for the Myo armband was lower at the beginning of the turn. As suggested by Figure 9b, the higher vehicle speed at the beginning of the turn resulted in lower ground reaction forces at the front steering tires, and thus less torque was applied by the DC motor to produce a lower SWR. On the other hand, Figure 9d and Figure 11d indicate that vehicle speed was lower towards the beginning of the turn could have resulted in a higher steering wheel rate, in the case of the wide U-turn.

When manually operating the steering wheel to perform the 45° turn, drivers applied the highest maximum and average SWRs (Table 1). Although the controller for the Myo armband had lower SWRs, it had higher path-following accuracy than the steering wheel. Since the SWR increases as more torque is inputted into the steering column, the lower SWRs of the Myo armband suggest that the steering wheel angle controller of the Myo armband uses less energy than manual steering wheel operation to achieve path-following accuracy during the 45° turn. This difference in energy usage could be due to driver behavior that produces excessive SWR.

A potential explanation for the decreasing average vehicle speed of the Myo armband through the 90° turn concerns the vehicle position at which the turn was initiated. As shown in Figure 9b, drivers tended to initiate turning later when using the Myo armband, instead of the steering wheel. Based on vehicle dynamics, the drivers needed to decelerate the vehicle in order to follow the road cones more closely. In accordance with the experimental results, the consequences of this deceleration were increased centripetal acceleration, lower average SWA, and higher maximum and average SWRs. However, this deceleration was not enough to compensate for decreased path-following accuracy. One remedy is to train drivers to initiate steering with the Myo armband earlier during the 90° turn. It is also possible that drivers will learn through experience and acquired trust in the Myo armband to initiate steering earlier.

On the other hand, in the cases of the 45° turn and the narrow U-turn, the Myo armband had higher path-following accuracy than the steering wheel. As shown in Figure 8a, the Myo armband was used to initiate turns at a vehicle position along the course that was slightly before the position for the steering wheel. Hence, the drivers did not need to decelerate, as in the case of the 90° turn. 

However, as indicated by Figure 8c, the initiation of the narrow U-turn with the Myo armband tended to be at a vehicle position slightly after the vehicle position for the steering wheel. Consequently, at approximately 10 s into the narrow U-turn, drivers had to decelerate the vehicle more when using the Myo armband, rather than the steering wheel (Figure 9c). Thus, increased centripetal acceleration for the Myo armband is apparent in Figure 10c. Despite the need to decelerate in the case of the narrow U-turn, the drivers used the Myo armband to steer the vehicle closer to the target trajectory for most of the turn. Hence, the path-following accuracy of the Myo armband was higher than for the steering wheel. 

With regard to the path-following accuracy of the wide U-turn, a previous driving simulator study conducted by the investigators had test drivers perform a wide U-turn that had twice the minimum vehicle turning radius, as in the current study [25]. This study predicted that a sEMG-based interface with a SWR that was similar to the SWR of the steering wheel would have comparable path-following accuracy, despite the ability of drivers to correct the vehicle trajectory when manually rotating the steering wheel. This correction is evident in Figure 8d, where the average trajectory of the steering wheel becomes increasingly more accurate throughout the turn. In contrast, path-following accuracy could have decreased because the Myo armband did not allow for such a correction. Unlike the previous driving simulator study, the Myo armband had lower path-following accuracy than the steering wheel.

### 4.2. Limitations

Section 4.1 explained that the path-following accuracy associated with the Myo armband for the 90° turn decreased due to the initiation of steering at a position further along the vehicle course than the position for the steering wheel. Hence, subsequent studies could train test drivers to initiate steering at a prior position to improve path-following accuracy. Alternatively, in order to produce a narrower trajectory that more accurately follows the 90° turn, it is possible reduce the response time of the Myo armband by increasing the SWR. In order to increase the SWR, the DC motor that receives commands from the Myo armband could be adjusted to provide a higher torque input to the steering column. Given that the Myo armband does not allow for steering corrections, additional torque input during the wide U-turn could also be applied to realize steering correction and path-following accuracy that is comparable to manual steering wheel operation.

As with other studies concerning automotive interfaces, a wider variety of interfaces could be compared to the sEMG-based interface, including haptic guidance, joysticks, BCIs, and power steering [9,43]. Further studies could also recruit a greater number and variety of test subjects who have hemiplegia or upper limb amputations, in order to evaluate human factors such as the level of trust in the Myo armband, sense of agency, task load, and perceived ease-of-use [44,45,46]. Although applicability of the proposed sEMG-based interface to drivers outside of the experiment is limited by the small number of test drivers who participated, the current experimental results demonstrate the possibility of one-handed remote steering wheel operation with sEMG signals. The applicability of the interface is also limited to low-speed turning maneuvers on residential roads, as reflected by the design of the driving scenarios. Future work will focus on more complicated maneuvers at higher speeds, in order to generalize the path-following accuracy or other performance metrics of the interface.

Increasing the variety and number of participants along with the variety of the driving scenarios would provide more empirical data, and therefore stronger statistical power for human factors studies. The evaluation of human factors could provide an explanation for driver behaviors such as deceleration during the wide U-turn and 90° turn and the tendency of Myo armband users in the current study to initiate steering later along the 90° turn, in contrast to the earlier initiation of the steering wheel.

## 5. Conclusions

Based on the performance of four turning maneuvers with an actual automobile, the path-following accuracy of the Myo armband was confirmed with respect to the steering wheel of the automobile. The Myo armband was superior in the case of the 45° turn and narrow U-turn. However, the steering wheel was more accurate in the case of the 90° turn and wide U-turn.

In order to improve the path-following accuracy of the Myo armband, steering could be initiated at an earlier vehicle position during the 90° turn or the SWR of the SWA controller for the Myo armband could be increased. Despite the possibility to improve the operation of the Myo armband, the results of the current study are significant because, unlike previous studies that only tested prototype sEMG-based steering assistance with driving simulations, the path-following accuracy of the mass-produced Myo armband was tested in multiple scenarios with an actual automobile. Despite previously mentioned limitations, the results of this study confirm that the Myo armband could be feasibly applied in future automobile tests.

## Figures and Tables

**Table 1:** Steering wheel rates of Myo armband and steering wheel

Interface	Driving Scenario	Average Steering Wheel Rate (°/s)	Standard Deviation of Steering Wheel Rate (°/s)	Maximum Steering Wheel Rate (°/s)
Steering wheel	45° turn	152.82	185.18	499.11
	90° turn	47.08	37.61	96.94
	Narrow U-turn	87.73	79.93	247.31
	Wide U-turn	18.32	9.44	34.04
Myo armband	45° turn	89.98	136.60	364.05
	90° turn	57.35	55.61	149.84
	Narrow U-turn	42.86	34.87	95.11
	Wide U-turn	20.13	13.80	44.62
